# Association between urbanisation and type 2 diabetes: an ecological study

**DOI:** 10.1136/bmjgh-2017-000473

**Published:** 2017-10-23

**Authors:** Zakariah Gassasse, Dianna Smith, Sarah Finer, Valentina Gallo

**Affiliations:** 1 Centre for Primary Care and Public Health, Blizard Institute, Queen Mary University of London, London, UK; 2 Faculty of Geography, University of Southampton, Southampton, UK; 3 Department of Epidemiology and Biostatistics, School of Public Health, Imperial College London, London, UK; 4 Epidemiology and Medical Statistic Unit, Faculty of Epidemiology and Population Health, London School of Hygiene & Tropical Medicine, London, UK

**Keywords:** environmental health, epidemiology, public health, descriptive study, nutrition

## Abstract

**Introduction:**

Previous studies have explored the effect of urbanisation on the prevalence of type 2 diabetes (T2D) at regional/national level. The aim of this study is to investigate the association between urbanisation and T2D at country level, worldwide, and to explore the role of intermediate variables (physical inactivity, sugar consumption and obesity). The potential effect modification of gross domestic product (GDP) was also assessed.

**Methods:**

Data for 207 countries were collected from accessible datasets. Direct acyclic graphs were used to describe the association between urbanisation, T2D and their intermediate variables (physical inactivity, sugar consumption and obesity). Urbanisation was measured as urban percentage (UP) and as agglomeration index (AI). Crude and multivariate linear regression analyses were conducted to explore selected associations. The interaction between urbanisation and T2D across levels of GDP per capita was investigated.

**Results:**

The association between urbanisation and T2D diverged by exposure: AI was positively associated, while UP negatively associated with T2D prevalence. Physical inactivity and obesity were statistically significantly associated with increased prevalence of T2D. In middle-income countries (MIC) UP, AI and GDP were significantly associated with T2D prevalence, while in high-income countries (HIC), physical inactivity and obesity were the main determinant of T2D prevalence.

**Conclusions:**

The type of urban growth, not urbanisation per se, predicted T2D prevalence at country level. In MIC, population density and GDP were the main determinant of diabetes, while in HIC. these were physical inactivity and obesity. Globalisation is playing an important role in the rise of T2D worldwide.

**Key questions**

**What is already known about this topic?**

- Urban environments are regarded as potentially obesogenic and diabetogenic.
- The majority of the studies investigating the association between urbanisation and diabetes found a positive association; however, it is not clear if this is a global trend, and if the mechanisms explaining the association are consistent across low-income and high-income countries, and therefore across different stages of the epidemiological and nutritional transition.

**What are the new findings?**

- This worldwide ecological analysis investigates the association between urbanisation and prevalence of diabetes, exploring the role of potentially mediating factors, that is, obesity, physical inactivity and sugar consumption.
- The present data suggest that it is the uncontrolled growth of large urban agglomerates, rather than urbanisation per se, which is associated to a higher prevalence of diabetes worldwide.
- Agglomeration index and gross domestic product per capita are the determinant of diabetes in upper-middle income countries, while in high-income countries, obesity and physical inactivity explain its prevalence.

**Recommendations for policy**

- The effect of urbanisation on diabetes prevalence differs depending on the stage of the epidemiological and nutritional transition countries are going through.
- A controlled and effective urbanisation can confer an ‘urban advantage’, which mitigates the inequalities associated to the rapid expansion of urban agglomerates.
- This would also counteract the surge of risk factors for chronic diseases limiting the non-communicable disease epidemic.

## Introduction

In 2015, the International Diabetes Federation reported that type 2 diabetes (T2D) was the fourth leading cause of death worldwide, with 415 million people affected.^1^ Existing literature has examined the contextual effects of urbanisation on T2D risk.^2 3^ A structural change from agriculture to industrialisation has reduced the cost of calories through agricultural innovation and by producing and processing energy-dense foods^4^; a recent study has identified changes in obesity prevalence following alternations to agriculture in India.^5^ Meanwhile, the cost of fruit and vegetables has increased due to the limited supply cultivated in less agriculturally productive land.^6^ Internal migration contributes to changes in industrial practices and has a role in changing health outcomes. As populations move towards a more urban environment, higher rates of obesity and T2D have been observed,^7^ likely as a consequence of changes in lifestyles and health behaviours (ie, diet and physical activity)^8–10^ but perhaps also due to the changing socioeconomic make-up of these new urban populations.

Moreover, increasingly, urban sprawl replaces green space with densely populated buildings, reducing outdoor spaces suitable for physical activity.^11^ This also hampers proximity and connectivity, where the increase in distance and time to make journeys discouraged society from walking or cycling.^12^ Economic literature shows that urban sprawl is more common in higher income countries (HIC) and that it is a proxy for affluence^13–15^; living in urban environment might also facilitate access to healthcare and preventive programmes.^16^ Few studies have examined the association between urbanisation and T2D at regional/national level finding mostly,^17–20^ but not always,^21^ higher prevalence in higher urbanised areas.

It is not clear to what extent urban growth per se is associated with higher prevalence of T2D, or a rapidly increasing urban concentration might promote an obesogenic or diabetogenic environment. Most measures of urbanisation in relation to non-communicable diseases were previously found of limited value in measuring the urbanisation process.^22^ The aim of this study is to investigate the association between urbanisation and T2D at country level, worldwide, and to examine the role of the main potentially modifiable lifestyle risk factors (physical inactivity, sugar consumption and obesity) in mediating this association. The potential effect modification of gross domestic product (GDP) was also explored.

## Methods

Data on the exposure variable (urbanisation), the outcome variable (prevalence of T2D) and potential intermediate and interacting variables or confounders (physical inactivity, prevalence of obesity, sugar consumption and GDP per capita) at country level, worldwide, were collected.

### Urbanisation

There is no consensus on how to measure urbanisation at country level; few indicators have been suggested, providing different proxy measures. Data on urbanisation measured by urban percentage (UP), that is, the proportion of a population living in urban areas as defined by national statistical offices, was collected for 207 countries from the 2015 World Bank Development Indicators.^23^ UP, despite being the most commonly used and widely available measure because of its simplicity, relies on country-specific definition of what it is urban, potentially leading to different ranks of urbanisation when several countries are considered. As a consequence, also data on the agglomeration index (AI) in 2008 was obtained for 162 countries from The World Bank World Development Report.^24^ AI is a composite measure of population density, size and travel time to the nearest urban city. Population density is based on the average of two global gridded population data sources—Global Rural-Urban Mapping Project and LandScan. Population size in a defined ‘large’ urban centre used for this analysis was 100 000 inhabitants. Travel time to the nearest urban city is calculated by a cost–distance model that estimates travel time to the city over the average travel speeds, based on GIS data, between the transport network and off road surfaces. These components are aggregated, with the proportion of this number to that country’s total population being the AI. This measure is designed to quantify the degree of settlement concentration in order to capture the difference between large cities growing bigger from many small cities emerging.^24^ Also, AI includes only locations that satisfy all three components, transcending country-specific and ad hoc definition discrete entities, such as cities and administrative boundaries.^25^ However, AI is sensitive to the chosen threshold values used to define the components.

### Type 2 diabetes mellitus prevalence

Prevalence of T2D was calculated using the 2015 World Bank Development Indicators^26^ reporting the percentage of people, aged 20–79 years, diagnosed with diabetes from 207 countries. These figures aggregated type 1 and type 2 diabetes; however, type 1 diabetes is, on average, a small proportion (up to 10%) of prevalent cases^27^; therefore, it is possible to use the aggregate measure to approximate T2D prevalence.

### Physical inactivity

Physical inactivity was derived from The 2010 WHO Global Health Observatory Data Repository^28^ for 143 countries as the proportion of a population, aged 20–79 years, achieving less than 150 min of moderate-intensity physical activity or less than 75 min of vigorous-intensity physical activity per week, reflecting current recommendations.

### Obesity prevalence

Prevalence of obesity was collected for 187 countries from the 2014 Central Intelligence Agency World Factbook^28^ as age-adjusted measure of the proportion of the population, aged 20–79 years, with a body mass index (BMI) of 30 kg/m^2^ or higher.

### Sugar consumption

Sugar and sweeteners consumption (kg/capita/year) is obtained from The UN Food and Agriculture Organisation Database^29^ for 173 countries and measures the supply in kilograms, for human consumption per year. This is calculated by dividing the annual sugar production by the mid-year population.

### GDP per capita

GDP per capita is the GDP divided by the mid-year population in US dollars. This was extracted from the World Bank^30^ 2015 for 183 countries and used as continuous variables (GDP per capita in $/1000) in multivariate models. Countries were also stratified by income groups based on the World Bank’s latest country classifications (2015) in low (<$1025), lower middle ($1026–$4035), upper middle ($4036–$12 475) and high (≥$12 476) income countries.^31^

### Statistical analysis

**Figure 1:**
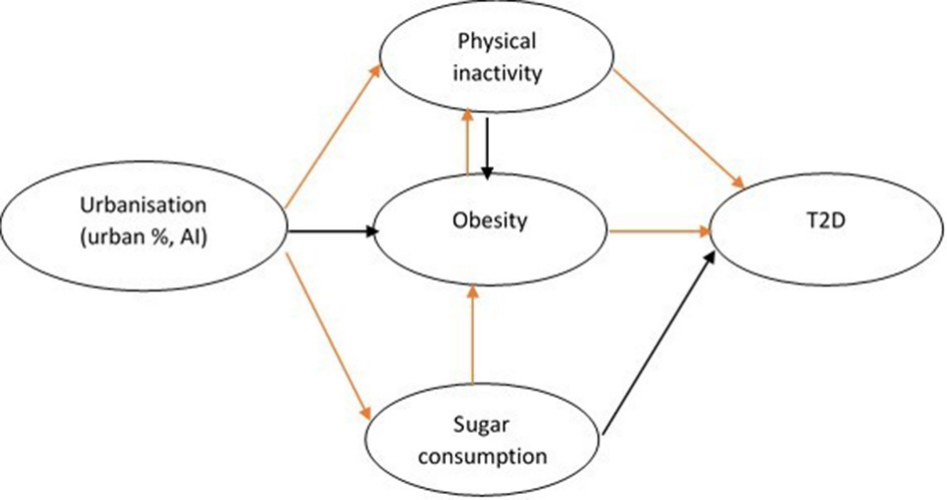
A conceptual framework disentangling the reciprocal associations of the variables used in the analysis, using the directed acyclic graph. In orange, the association that were found significant statistically in the multivariate models (table 1). AI, agglomeration index; T2D, type 2 diabetes.

A conceptual framework for studying the interconnectedness of the variables included in this study was built using directed acyclic graphs (DAGs) (figure 1): measures of urbanisation are regarded as the main exposure variables; obesity, physical inactivity and sugar consumption as intermediate variables; and prevalence of T2D as the outcome variable. Accordingly, crude associations of each suitable variable (exposure or intermediate variables) with T2D and with intermediate variables were initially studied. Subsequently, few multivariate models were built to study the association between urbanisation (measured either as UP or as AI) and intermediate variables, with GDP and other relevant variables (ie, physical inactivity when studying obesity and vice versa) as potential confounders. Finally, a model including UP, the AI and intermediate variables was built. Interaction of the association between urbanisation and T2D across income categories has been tested using the likelihood ratio test; the final model was repeated restricted to each of the income categories. All analyses were conducted using STATA V.14.1. Statistical significance was set at p<0.05.

## Results

**Figure 2:**
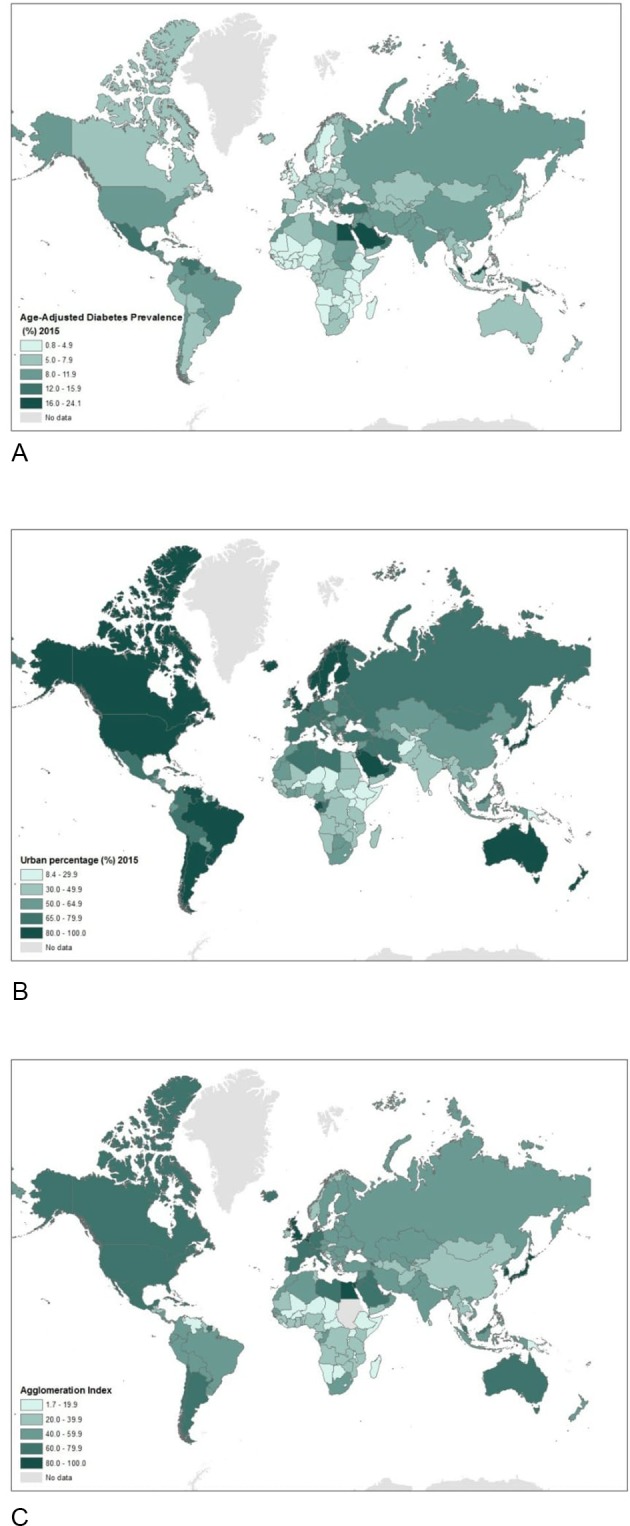
Maps showing the age-adjusted prevalence of T2D as proportion of the total population aged 20–79 years in 2015, worldwide (A); urban proportion, as proportion of total population at country level living in urban areas, worldwide (B); agglomeration index at country levels, worldwide (C). T2D, type 2 diabetes.

The age-adjusted prevalence of T2D at country level, the proportion of population living urban areas (or UP), and the AI at country level, worldwide, are shown in figure 2A–C. Statistical analyses include a maximum of 207 countries (crude association between urbanisation and T2D) and a minimum of 109 countries (multivariate analyses) allowing for missing values (table 1).

**Table 1:** Linear regression coefficients and relative 95% CIs coming from crude models and from multivariate models investigating the association of independent variables in relation to T2D and intermediate variables (n=number of countries)

Variates	Crude β coefficient (95% CI)			Adjusted (urban percentage) β coefficient (95% CI)		Adjusted (agglomeration index) β coefficient (95% CI)
	N	T2D	N	T2D	N	T2D
Urban percentage	207	0.048* (0.022 to 0.074)	126	−0.024 (−0.058 to 0.009)	–	–
Agglomeration index	162	0.082* (0.058 to 0.105)	–	–	109	0.054* (0.019 to 0.089)
Obesity	187	0.281* (0.233 to 0.329)	126	0.233* (0.149 to 0.317)	109	0.148* (0.052 to 0.244)
Physical inactivity	143	0.204* (0.142 to 0.265)	126	0.142* (0.085 to 0.199)	109	0.106* (0.045 to 0.167)
Sugar consumption	173	0.114* (0.075 to 0.152)	126	0.016 (−0.044 to 0.076)	109	0.011 (−0.056 to 0.078)
GDP per capita	183	0.020 (−0.018 to 0.057)	126	−0.060 (−0.103 to −0.017)	109	−0.069 (−0.108 to −0.031)
	N	Physical inactivity	N	Physical inactivity	N	Physical inactivity
Urban percentage	143	0.210* (0.136 to 0.283)	136	0.128* (0.033 to 0.224)	–	–
Agglomeration index	118	0·261* (0.185 to 0.336)	–	–	112	0.203* (0.111 to 0.295)
Obesity	141	0.461* (0.305 to 0.617)	136	0.359* (0.185 to 0.532)	112	0.216 (−0.006 to 0.437)
GDP per capita	138	0.182* (0.080 to 0.285)	136	−0.007 (−0.126 to 0.111)	112	0.025 (−0.082 to 0.132)
	N	Obesity	N	Obesity	N	Obesity
Urban percentage	187	0.229* (0.170 to 0.288)	126	0.045 (−0.027 to 0.117)	–	–
Agglomeration index	153	0.234* (0.177 to 0.290)	–	–	109	0.040 (−0.031 to 0.110)
Physical inactivity	141	0.429* (0.284 to 0.574)	126	0.112 (−0.008 to 0.233)	109	0.109 (−0.012 to 0.231)
Sugar consumption	166	0.457* (0·388 to 0.525)	126	0.432* (0.328 to 0.535)	109	0.426* (0.317 to 0.534)
GDP per capita	178	0.236* (0.151 to 0.321)	126	−0.015 (−0.107 to 0.077)	109	0.020 (−0.059 to 0.981)
	N	Sugar consumption	N	Sugar consumption	N	Sugar consumption
Urban percentage	173	0.370* (0.283 to 0.457)	165	0.242* (0·.136 to 0.347)	–	–
Agglomeration index	148	0.375* (0.282 to 0.468)	–	–	143	0.280* (0.186 to 0.373)
GDP per capita	165	0.451* (0.339 to 0.563)	165	0.273* (0.141 to 0.404)	143	0.305* (0.193 to 0.417)

*p Value <0.005.

GDP, gross domestic product; T2D, type 2 diabetes.

Crude linear regression models assessing the association between the independent variables and T2D prevalence are shown in table 1. Increasing urbanisation is associated with increasing T2D prevalence regardless of the variable used: UP (β=0.048, 95% CI 0.022 to 0.047) or AI (β=0.082, 95% CI 0.058 to 0.105). Contrary to GDP, physical inactivity, obesity and sugar consumption are univariately significantly associated with T2D.

Urbanisation, obesity and GDP were all positively associated with physical inactivity in a statistically significant way. Similarly, urbanisation, physical inactivity, sugar consumption and GDP were positively significantly associated with obesity. Urbanisation and GDP were also significantly associated with sugar consumption (table 1).

In multivariate analyses, urbanisation measured either as UP or AI was found significantly and positively associated with sugar consumption and physical inactivity, but not with obesity. In turn, higher sugar consumption was significantly associated with higher obesity, and higher obesity was significantly associated with higher physical inactivity (table 1 and figure 1, DAGs connectors in orange).

When all the variables were included in a final model, higher obesity prevalence, higher levels of physical inactivity and lower GDP were all significantly associated with higher prevalence of T2D, entirely accounting for the association between UP and T2D. Nonetheless, when urbanisation was measured by the AI, this remained positively statistically significantly associated with T2D in the final model and so did the intermediate variables (physical inactivity, obesity and GDP) (table 1).

A statistically significant interaction was observed between urbanisation measured either as UP or AI and T2D prevalence across categories of country income (p=0.04 and p<0.001, respectively). When UP and AI where added to the same model, the former was estimated to be significantly negatively associated with T2D (β=-0.047, 95% CI −0.088 to −0.005), while latter was significantly positively associated (β=0.066, 95% CI 0.030 to 0.102) (table 2). When stratified by income categories, these associations were still significant only in middle-income countries (table 2).

**Table 2:** Linear regression coefficients and relative 95% CIs coming from multivariate models across the four categories of GDP (n=number of countries)

	All countries β coefficient (95% CI)	Low income β coefficient (95% CI)	Lower middle income β coefficient (95% CI)	Upper middle income β coefficient (95% CI)	High income β coefficient (95% CI)
	n=126	n=21	n=32	n=29	n=44
Diabetes prevalence, median % (IQR)	6.6 (4.4 to 9.8)	3.4 (2.2 to 4.1)	6.5 (3.7 to 8.2)	9.9 (8.8 to 11.7)	7.1 (5.4 to 10.0)
Urban percentage	−0.047* (−0.088 to −0.005)	0.038 (−0.086 to 0.163)	−0.078 (−0.158 to 0.002)	−0.140* (−0.241 to −0.039)	−0.018 (−0.077 to 0.041)
Agglomeration index	0.066* (0.030 to 0.102)	−0.031 (−0.185 to 0.123)	0.087* (0.008 to 0.166)	0.108* (0.032 to 0.184)	0.028 (−0.025 to 0.082)
Obesity	0.177* (0.079 to 0.275)	−0.606 (−1.535 to 0.323)	0.071 (−0.115 to 0.258)	−0.004 (−0.261 to 0.253)	0.247* (0.091 to 0.402)
Physical inactivity	0.115* (0.054 to 0.175)	0.011 (−0.098 to 0.119)	0·.109 (−0.078 to 0.297)	−0.009 (−0.121 to 0.102)	0.200* (0.097 to 0.302)
Sugar consumption	0.014 (−0.052 to 0.080)	0.137 (−0.089 to 0.364)	0.104 (−0.009 to 0.218)	0.081 (−0.055 to 0.216)	−0.063 (−0.171 to 0.046)
GDP	−0.051* (−0.093 to −0.010)	−0.202 (−5.974 to 5·571)	0.310 (−0.765 to 1.385)	0.776* (0.041 to 1.512)	−0.015 (−0.071 to 0.400)

*p Values <0.005.

GDP, gross domestic product.

The association between urbanisation, intermediate variables and T2D varied across income categories: while none of the variables is associated with T2D prevalence in lower income countries, the AI is the only variable significantly positively associated with T2D in lower income and middle-income countries (LMIC), irrespective of intermediate variable levels. Upper middle-income countries with higher prevalence of T2D have higher AI, lower UP and higher GDP. Finally, in HIC only high prevalence of obesity and physical inactivity are associated with higher prevalence of T2D, irrespective of GDP and urbanisation (table 2).

## Discussion

This is an ecological analysis investigating the determinants of the association between urbanisation and T2D globally. While the proportion of people living in urban settings (UP) is, if anything, negatively associated with the prevalence of T2D, a measure of urban concentration (AI) is strongly positively associated with T2D prevalence. These associations are mediated, though not entirely explained, by the prevalence of some known risk factors for T2D (obesity and physical inactivity) and by lower GDP. Sugar consumption at country level is a determinant of obesity prevalence but is not associated per se to T2D prevalence. In turn, obesity is associated with physical inactivity, and both are then associated with increased prevalence of T2D.

Notably, urbanisation measured as UP or as AI has inconsistent effects on the risk of T2D. UP is based on member countries’ existing definitions of what constitutes an urban or a rural area. Not only do these definitions differ widely by country, in many places the traditional urban/rural dichotomy is becoming increasingly inadequate.^32^ The fact that this measure was negatively associated with T2D prevalence in the final model might therefore reflect the ‘urban advantage’ paradigm, where economic development, through urbanisation, improves social welfare.^3^ Having urban communities better spatial access to welfare facilities that offer preventative care contributes to increasing preventability of chronic diseases such as T2D.^33^ However, the AI, distinguishing between large urban cities that grow fast leading to highly densely populated areas and the emergence of new, manageable urban agglomerates, accounts—at least partially—for the ‘urban advantage’ effect, and it is significantly positively associated with T2D prevalence.

The analysis by income categories suggests how the socioeconomic and lifestyle factors can prevail in modulating the risk of T2D across different stages of the nutritional transition these countries are going through. During the early phases of the transitions experienced by LMIC, agglomeration density measured by the AI is the only factor found to be associated with increased T2D prevalence. While the transition progresses, other socioeconomic factors, including GDP, play an important role in determining the risk of T2D, regardless of its lifestyle risk factor distribution (upper middle income countries (UMIC)). In HIC, by the end of the transition, lifestyle risk factors such as obesity and physical inactivity are associated with T2D prevalence, regardless of the socioeconomic ones and urbanisation. This suggest that during the transition, a plethora of complex risk factors cluster according to socioeconomic variables so strongly as to making the individual effect of some of them negligible. Living in highly dense, overcrowded urban conditions that could increase the risk of T2D and other non-communicable diseases reflects the ‘urban penalty’ where, historically, low socioeconomic groups are spatially situated in concentric zones, exposing them to poorer lifestyle habits as well as poorer access to healthcare, sanitation and healthy nutritional options.^34^

Findings from individual studies mainly support this trend. Among LMIC, recent findings from India showed a higher prevalence of T2D in low socioeconomic groups living in the rapidly expanding urban areas of the more economically developed states.^35^ Among UMIC, data from Peru show how mortality and diabetes-specific mortality is higher in urban compared with rural context; internal migrants from rural to urban areas maintain their lower mortality risk, probably reflecting a healthy cohort effect.^36^ However, the longer migrants live in the new urban context, the higher their risk of becoming obese.^37^ A multilevel analysis conducted in China, finding double diabetes prevalence in highly urban areas compared with rural ones, suggests that community economic factors, modern markets, communications and transportation infrastructure might be responsible for the differences.^17^ In HIC, data coming from Oman, showed how diabetes, obesity, hypertension and high cholesterol were more prevalent in urban areas compared with rural ones.^19^ In Greenland, among Inuits, diabetes was more prevalent in small town than big cities despite people in small town were found to be exercising more but also smoking more.^20^

The nutritional transition experienced at different levels by countries worldwide is strongly associated with the process of globalisation. Globalisation is associated with changing incomes and lifestyles, and it alters the quantity, type, cost and desirability of foods available for consumption by altering the nature of agrifood systems.^38^ The ways globalisation impacts the nutritional transition are multiple and interconnected, for example, food trade and global sourcing, foreign direct investment, global advertising and retail reconstructing. However, determining which are the specific mechanisms by which globalisation alters diet quality and quantity is a challenge, and assessing their long-term health effect is even more complicated.^39 40^ Urbanisation has a pivotal role in the effect of globalisation on the nutritional transition, with the rise of multinational supermarkets and fast food chains expanding beyond urbanised areas and into smaller cities and towns. This allows producers to mobilise the supply of energy-dense foods and calorically sweetened beverages to new markets at cheap prices.^41^ Urbanisation is also associated with occupation-related physical inactivity in the administrative and services sector that are incompatible with home food production and consumption, exacerbated by increasing infrastructures and transport networks that limit land available for cultivation and recreation.^42^ However, the effects of urbanisation on lifestyle are not immediate; most studies explore changes to health of rural to urban migrants over years to assess the impact of internal migration on health behaviours.^7^ Urban planning for increasing population health is becoming an emerging global challenge; eight interventions designed to encourage walking, cycling and public transport use while reducing private motor vehicle use were proposed, including equitable distribution of employment across cities, designing pedestrian-friendly and cycling-friendly movement networks, reducing distance to public transport and enhancing the desirability of active travel modes.^43^ However, this needs to be coupled to an adequate access to healthcare and health education and a minimised westernisation of diet in countries undergoing the nutritional transition.^44^

The main limitation of this study is its ecological design, which does not allow generalisation of the associations found at country level to an individual level (ecological fallacy).^45^ However, the present results are in line with previous ecological and observational study findings, indirectly arguing against ecological fallacy. An ecological study investigating dietary patterns in association with diabetes prevalence found an increasing prevalence across agricultural/transitional/westernised nutrition patters which, in turn, were associated with increasing urbanisation and decreasing physical activity.^46^ A systematic review of individual-based studies in Southeast Asia was also highly coherent with the present ecological findings: point estimates of the associations between urbanisation and diabetes coming from single studies were not poolable due to high heterogeneity. However, the association was shown to be modified by per-capita gross national income of the country where the study took place being much stronger in countries with a lower gross national income.^47^

Other limitations include genetic population stratification, which could confound the results, with some ethnic groups (eg, South Asians) at higher genetic risk of T2D.^48 49^ Ethnicity-specific cut-offs of BMI were not used and therefore may underestimate obesity prevalence in some populations. A residual confounding effect could also be due to diet or other unmeasured variables. Finally, the analysis did not take directly into account access to healthcare, internal and external migration, literacy levels and education, which might account for some of the differences in T2D prevalence. The role of some of these factors might be particularly difficult to explore as they might lay on the causal pathway linking urbanisation with T2D prevalence (ie, migration or access to healthcare).

## Conclusion

The uncontrolled rapid growth of highly densely populated urban agglomerates is associated with an increased prevalence of T2D worldwide. In UMIC, which have nearly completed the epidemiological and nutritional transition, more densely urbanised and relatively richer economies have higher prevalence of T2D. In HIC, the prevalence of T2D is not associated with wealth nor urbanisation but with a more diabetogenic environment characterised by higher prevalence of obesity and physical inactivity. An urbanisation process effectively targeting urban planning, including access to healthcare, health inequalities associated to the rapid expansion of urban agglomerates; and the risks associated to the westernisation of the diet might be effective in limiting the non-communicable disease epidemic in countries undergoing the epidemiological and nutritional transition.
